# Na_3_DyCl_6_

**DOI:** 10.1107/S1600536811014498

**Published:** 2011-04-29

**Authors:** Christian M. Schurz, Gerd Meyer, Thomas Schleid

**Affiliations:** a Institut für Anorganische Chemie, Universität Stuttgart, Pfaffenwaldring 55, 70569 Stuttgart, Germany; b Department für Chemie, Institut für Anorganische Chemie, Universität zu Köln, Greinstrasse 6, 50939 Köln, Germany

## Abstract

Single crystals of the title compound, tris­odium hexa­chloridodysprosate, Na_3_DyCl_6_, were obtained as a by-product of synthesis using dysprosium(III) chloride and sodium chloride among others. The monoclinic structure with its typical β angle close to 90° [90.823 (4)°] is isotypic with the mineral *cryolite* (Na_3_AlF_6_) and the high-temperature structure of the Na_3_ *M*Cl_6_ series, with *M* = Eu–Lu, Y and Sc. The isolated, almost perfect [DyCl_6_]^3−^ octa­hedra are inter­connected *via* two crystallographically different Na^+^ cations: while one Na^+^ resides on centres of symmetry (as well as Dy^3+^) and also builds almost perfect, isolated [NaCl_6_]^5−^ octa­hedra, the other Na^+^ is surrounded by seven chloride anions forming a distorted [NaCl_7_]^6−^ trigonal prism with just one cap as close secondary contact.

## Related literature

The first structural descriptions of the Na_3_ *M*Cl_6_ series (*M* = Eu–Lu, Y and Sc) on a single crystal in the *cryolite*-type structure (Hawthorne &, Ferguson, 1975 ▶) were given for *M* = Er by Meyer *et al.* (1987 ▶), for *M* = Ho by Böcker *et al.* (2001 ▶) and for *M* = Y by Liao & Dronskowski (2004 ▶). For the correlation between the two temperature-dependent phases, see: Meyer (1984 ▶); Meyer *et al.* (1987 ▶); Wickleder & Meyer (1995 ▶). For a planned synthesis of Dy_2_NCl_3_, compare with those for Gd_2_NCl_3_ (Schwanitz-Schüller & Simon, 1985 ▶) and Y_2_NCl_3_ (Meyer *et al.*, 1989 ▶).

## Experimental

### 

#### Crystal data

Na_3_DyCl_6_
*M* *_r_* = 444.17
Monoclinic, 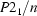

*a* = 6.8791 (5) Å
*b* = 7.2816 (5) Å
*c* = 10.1734 (7) Å
β = 90.823 (4)°
*V* = 509.54 (6) Å^3^
*Z* = 2
Mo *K*α radiation
μ = 8.96 mm^−1^
*T* = 293 K
0.20 × 0.15 × 0.10 mm

#### Data collection

Nonius Kappa-CCD diffractometer
Absorption correction: numerical (*X-SHAPE*; Stoe & Cie 1999 ▶) *T*_min_ = 0.218, *T*_max_ = 0.414
12026 measured reflections
1245 independent reflections
1124 reflections with *I* > 2σ(*I*)
*R*_int_ = 0.071

#### Refinement

*R*[*F* ^2^ > 2σ(*F* ^2^)] = 0.019
*wR*(*F* ^2^) = 0.045
*S* = 1.08
1245 reflections
50 parameters
Δρ_max_ = 0.84 e Å^−3^
Δρ_min_ = −1.05 e Å^−3^

### 

Data collection: *COLLECT* (Nonius, 1998 ▶); cell refinement: *SCALEPACK* (Otwinowski & Minor, 1997 ▶); data reduction: *SCALEPACK* and *DENZO* (Otwinowski & Minor, 1997 ▶); program(s) used to solve structure: *SHELXS97* (Sheldrick, 2008 ▶); program(s) used to refine structure: *SHELXL97* (Sheldrick, 2008 ▶); molecular graphics: *DIAMOND* (Brandenburg, 2006 ▶); software used to prepare material for publication: *SHELXL97*.

## Supplementary Material

Crystal structure: contains datablocks I, global. DOI: 10.1107/S1600536811014498/hp2006sup1.cif

Structure factors: contains datablocks I. DOI: 10.1107/S1600536811014498/hp2006Isup2.hkl

Additional supplementary materials:  crystallographic information; 3D view; checkCIF report

## Figures and Tables

**Table 1:** Selected bond lengths (Å)

Na1—Cl2^i^	2.7358 (8)
Na1—Cl3^iii^	2.7902 (8)
Na1—Cl1	2.8687 (8)
Na2—Cl1	2.8295 (19)
Na2—Cl2^vi^	2.8341 (19)
Na2—Cl1^iv^	2.8492 (19)
Na2—Cl3^i^	2.8612 (19)
Na2—Cl3^vii^	3.204 (2)
Na2—Cl2^iv^	3.325 (2)
Na2—Cl2	3.488 (2)
Dy—Cl2	2.6176 (8)
Dy—Cl3	2.6320 (8)
Dy—Cl1^viii^	2.6447 (8)

Symmetry codes: (i) 
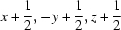
; (iii) 
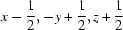
; (iv) 
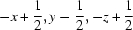
; (vi) 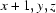; (vii) 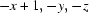; (viii) 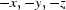.

**Fig. 1.**
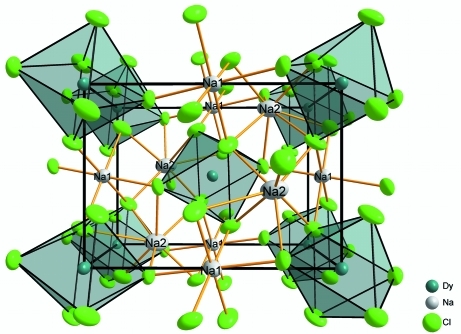
Crystal structure of cryolite-type Na3DyCl6. Displacement ellipsoids are drawn at 90% probability level.

## supplementary crystallographic information

### Comment

Trisodiumhexachlorodysprosate(III) belongs to a group of ternary chlorides Na_3_*M*Cl_6_ with *M* = Eu – Lu, Y and Sc (Meyer *et al.*, 1987), which crystallize in the *cryolite*-type structure (Hawthorne *et al.*, 1975). The Dy^3+^ and (Na1)^+^ occupy the 2*a* and 2*b Wyckoff* positions at centres of symmetry, whereas the three crystallographically different chloride anions and (Na2)^+^ reside at the 4*e* position with the site symmetry 1. A l l cations have six primary contacts to Cl^-^, but the [(Na2)Cl_6_]^5-^ polyhedron can not only be described as distorted trigonal prism instead of the usual octahedra that are realised for [DyCl_6_]^3-^ and [(Na1)Cl_6_]^5-^, it moreover carries a seventh capping Cl^-^ anion. The isolated [DyCl_6_]^3-^ octahedra are interconnected to a three-dimensional texture *via* sodium cations (Fig. 1). This structure represents the high-temperature phase of the Na_3_*M*Cl_6_ series with *M* = Eu – Lu, Y and Sc. The transition into the low-temperature phase with its trigonal structure (Meyer, 1984) depends on the radius of the actual lanthanoid(III) cation (Wickleder *et al.*, 1995) and is estimated for *M* = Dy at around 290 K, hence not far below the temperature of the measurement.

### Experimental

Colourless and transparent single crystals of Na_3_DyCl_6_ were obtained as by-product from the reaction of sodium azide (NaN_3_), dysprosium metal (Dy) and its the corresponding trichloride (DyCl_3_) in presence of sodium chloride (NaCl) as flux, originally designed to produce Dy_2_NCl_3_ in analogy to Gd_2_NCl_3_ (Schwanitz-Schüller *et al.*, 1985) and Y_2_NCl_3_ (Meyer *et al.*, 1989) instead. The reaction mixture was placed into a torch- sealed evacuated fused-silica vessel, which was heated at 1143 K for seven days, followed by cooling to room temperature within one day.

### Crystal data

**Table:**

Na_3_DyCl_6_	*F*(000) = 402
*M**_r_* = 444.17	*D*_x_ = 2.895 Mg m^−^^3^
Monoclinic, *P*2_1_/*n*	Mo *K*α radiation, λ = 0.71069 Å
Hall symbol: -p2yn	Cell parameters from 8457 reflections
*a* = 6.8791 (5) Å	θ = 3.4–28.1°
*b* = 7.2816 (5) Å	µ = 8.96 mm^−^^1^
*c* = 10.1734 (7) Å	*T* = 293 K
β = 90.823 (4)°	Block, colourless
*V* = 509.54 (6) Å^3^	0.20 × 0.15 × 0.10 mm
*Z* = 2	

### Data collection

**Table:**

Nonius KappaCCD diffractometer	1245 independent reflections
Radiation source: fine-focus sealed tube	1124 reflections with *I* > 2σ(*I*)
graphite	*R*_int_ = 0.071
charge cpouled device scans	θ_max_ = 28.1°, θ_min_ = 3.4°
Absorption correction: numerical (*X-SHAPE*; Stoe & Cie 1999)	*h* = −9→9
*T*_min_ = 0.218, *T*_max_ = 0.414	*k* = −9→9
12026 measured reflections	*l* = −13→13

### Refinement

**Table:**

Refinement on *F*^2^	Primary atom site location: structure-invariant direct methods
Least-squares matrix: full	Secondary atom site location: difference Fourier map
*R*[*F*^2^ > 2σ(*F*^2^)] = 0.019	*w* = 1/[σ^2^(*F*_o_^2^) + (0.0199*P*)^2^ + 0.2881*P*] where *P* = (*F*_o_^2^ + 2*F*_c_^2^)/3
*wR*(*F*^2^) = 0.045	(Δ/σ)_max_ < 0.001
*S* = 1.08	Δρ_max_ = 0.84 e Å^−^^3^
1245 reflections	Δρ_min_ = −1.05 e Å^−^^3^
50 parameters	Extinction correction: *SHELXL97* (Sheldrick, 2008), Fc^*^=kFc[1+0.001xFc^2^λ^3^/sin(2θ)]^-1/4^
0 restraints	Extinction coefficient: 0.0043 (5)

### Special details

**Table:**

Geometry. All e.s.d.'s (except the e.s.d. in the dihedral angle between two l.s. planes) are estimated using the full covariance matrix. The cell e.s.d.'s are taken into account individually in the estimation of e.s.d.'s in distances, angles and torsion angles; correlations between e.s.d.'s in cell parameters are only used when they are defined by crystal symmetry. An approximate (isotropic) treatment of cell e.s.d.'s is used for estimating e.s.d.'s involving l.s. planes.
Refinement. Refinement of *F*^2^ against ALL reflections. The weighted *R*-factor *wR* and goodness of fit *S* are based on *F*^2^, conventional *R*-factors *R* are based on *F*, with *F* set to zero for negative *F*^2^. The threshold expression of *F*^2^ > σ(*F*^2^) is used only for calculating *R*-factors(gt) *etc*. and is not relevant to the choice of reflections for refinement. *R*-factors based on *F*^2^ are statistically about twice as large as those based on *F*, and *R*- factors based on ALL data will be even larger.

### Fractional atomic coordinates and isotropic or equivalent isotropic displacement parameters (Å^2^)

**Table:**

	*x*	*y*	*z*	*U*_iso_*/*U*_eq_	
Na1	0.0000	0.0000	0.5000	0.0291 (4)	
Na2	0.5218 (2)	−0.0749 (2)	0.24225 (18)	0.0499 (4)	
Dy	0.0000	0.0000	0.0000	0.01593 (9)	
Cl1	0.13816 (12)	0.06522 (12)	0.23941 (8)	0.02834 (18)	
Cl2	−0.31489 (12)	0.17894 (12)	0.06382 (9)	0.0358 (2)	
Cl3	0.16836 (13)	0.30521 (11)	−0.07742 (9)	0.0358 (2)	

### Atomic displacement parameters (Å^2^)

**Table:**

	*U*^11^	*U*^22^	*U*^33^	*U*^12^	*U*^13^	*U*^23^
Na1	0.0323 (11)	0.0244 (10)	0.0303 (10)	−0.0038 (7)	−0.0033 (9)	0.0027 (7)
Na2	0.0462 (9)	0.0341 (9)	0.0694 (12)	−0.0069 (7)	0.0029 (8)	0.0033 (8)
Dy	0.01762 (12)	0.01512 (12)	0.01509 (12)	−0.00060 (6)	0.00126 (7)	−0.00138 (6)
Cl1	0.0334 (4)	0.0333 (4)	0.0182 (3)	0.0032 (3)	−0.0037 (3)	−0.0035 (3)
Cl2	0.0322 (4)	0.0323 (4)	0.0431 (5)	0.0149 (3)	0.0120 (4)	0.0090 (4)
Cl3	0.0350 (4)	0.0271 (4)	0.0450 (5)	−0.0123 (3)	−0.0069 (4)	0.0122 (4)

### Geometric parameters (Å, °)

**Table:**

Na1—Cl2^i^	2.7358 (8)	Na2—Dy^iv^	4.0608 (17)
Na1—Cl2^ii^	2.7358 (8)	Na2—Na1^x^	4.2126 (17)
Na1—Cl3^iii^	2.7902 (8)	Dy—Cl2	2.6176 (8)
Na1—Cl3^iv^	2.7902 (8)	Dy—Cl2^xii^	2.6176 (8)
Na1—Cl1	2.8687 (8)	Dy—Cl3	2.6320 (8)
Na1—Cl1^v^	2.8687 (8)	Dy—Cl3^xii^	2.6320 (8)
Na1—Na2^vi^	3.9579 (18)	Dy—Cl1^xii^	2.6447 (8)
Na1—Na2^vii^	3.9580 (18)	Dy—Cl1	2.6447 (8)
Na1—Na2^viii^	4.2126 (17)	Dy—Na2^xiii^	4.0608 (17)
Na1—Na2^ix^	4.2126 (17)	Dy—Na2^vi^	4.0608 (17)
Na2—Cl1	2.8295 (19)	Cl1—Na2^vi^	2.8492 (19)
Na2—Cl2^x^	2.8341 (19)	Cl2—Na1^xiv^	2.7358 (8)
Na2—Cl1^iv^	2.8492 (19)	Cl2—Na2^viii^	2.8342 (19)
Na2—Cl3^i^	2.8612 (19)	Cl2—Na2^vi^	3.325 (2)
Na2—Cl3^xi^	3.204 (2)	Cl2—Na2	3.488 (2)
Na2—Cl2^iv^	3.325 (2)	Cl3—Na1^vi^	2.7902 (8)
Na2—Cl2	3.488 (2)	Cl3—Na2^xv^	2.8611 (19)
Na2—Na1^iv^	3.9580 (18)	Cl3—Na2^xi^	3.204 (2)
			
Cl2^i^—Na1—Cl2^ii^	180.0	Cl3^xi^—Na2—Na1^iv^	44.32 (3)
Cl2^i^—Na1—Cl3^iii^	90.49 (3)	Cl2^iv^—Na2—Na1^iv^	88.01 (4)
Cl2^ii^—Na1—Cl3^iii^	89.51 (3)	Cl1—Na2—Dy^iv^	103.79 (5)
Cl2^i^—Na1—Cl3^iv^	89.51 (3)	Cl2^x^—Na2—Dy^iv^	158.34 (6)
Cl2^ii^—Na1—Cl3^iv^	90.49 (3)	Cl1^iv^—Na2—Dy^iv^	40.43 (3)
Cl3^iii^—Na1—Cl3^iv^	180.0	Cl3^i^—Na2—Dy^iv^	97.19 (5)
Cl2^i^—Na1—Cl1	85.33 (3)	Cl3^xi^—Na2—Dy^iv^	88.29 (4)
Cl2^ii^—Na1—Cl1	94.67 (3)	Cl2^iv^—Na2—Dy^iv^	39.98 (2)
Cl3^iii^—Na1—Cl1	86.30 (3)	Na1^iv^—Na2—Dy^iv^	78.73 (3)
Cl3^iv^—Na1—Cl1	93.71 (3)	Cl1—Na2—Na1^x^	132.81 (6)
Cl2^i^—Na1—Cl1^v^	94.67 (3)	Cl2^x^—Na2—Na1^x^	90.06 (4)
Cl2^ii^—Na1—Cl1^v^	85.33 (3)	Cl1^iv^—Na2—Na1^x^	112.16 (5)
Cl3^iii^—Na1—Cl1^v^	93.71 (3)	Cl3^i^—Na2—Na1^x^	41.17 (3)
Cl3^iv^—Na1—Cl1^v^	86.29 (3)	Cl3^xi^—Na2—Na1^x^	82.78 (4)
Cl1—Na1—Cl1^v^	180.00 (3)	Cl2^iv^—Na2—Na1^x^	40.45 (2)
Cl2^i^—Na1—Na2^vi^	59.53 (3)	Na1^iv^—Na2—Na1^x^	120.74 (4)
Cl2^ii^—Na1—Na2^vi^	120.47 (3)	Dy^iv^—Na2—Na1^x^	74.49 (3)
Cl3^iii^—Na1—Na2^vi^	53.35 (3)	Cl2—Dy—Cl2^xii^	180.0
Cl3^iv^—Na1—Na2^vi^	126.65 (3)	Cl2—Dy—Cl3	91.33 (3)
Cl1—Na1—Na2^vi^	45.99 (3)	Cl2^xii^—Dy—Cl3	88.67 (3)
Cl1^v^—Na1—Na2^vi^	134.01 (3)	Cl2—Dy—Cl3^xii^	88.67 (3)
Cl2^i^—Na1—Na2^vii^	120.47 (3)	Cl2^xii^—Dy—Cl3^xii^	91.33 (3)
Cl2^ii^—Na1—Na2^vii^	59.53 (3)	Cl3—Dy—Cl3^xii^	180.0
Cl3^iii^—Na1—Na2^vii^	126.65 (3)	Cl2—Dy—Cl1^xii^	91.73 (3)
Cl3^iv^—Na1—Na2^vii^	53.35 (3)	Cl2^xii^—Dy—Cl1^xii^	88.27 (3)
Cl1—Na1—Na2^vii^	134.01 (3)	Cl3—Dy—Cl1^xii^	91.71 (3)
Cl1^v^—Na1—Na2^vii^	45.99 (3)	Cl3^xii^—Dy—Cl1^xii^	88.29 (3)
Na2^vi^—Na1—Na2^vii^	180.0	Cl2—Dy—Cl1	88.28 (3)
Cl2^i^—Na1—Na2^viii^	127.96 (3)	Cl2^xii^—Dy—Cl1	91.72 (3)
Cl2^ii^—Na1—Na2^viii^	52.04 (3)	Cl3—Dy—Cl1	88.29 (3)
Cl3^iii^—Na1—Na2^viii^	42.45 (3)	Cl3^xii^—Dy—Cl1	91.71 (3)
Cl3^iv^—Na1—Na2^viii^	137.55 (3)	Cl1^xii^—Dy—Cl1	180.00 (3)
Cl1—Na1—Na2^viii^	73.28 (3)	Cl2—Dy—Na2^xiii^	125.31 (3)
Cl1^v^—Na1—Na2^viii^	106.72 (3)	Cl2^xii^—Dy—Na2^xiii^	54.69 (3)
Na2^vi^—Na1—Na2^viii^	72.02 (3)	Cl3—Dy—Na2^xiii^	115.46 (3)
Na2^vii^—Na1—Na2^viii^	107.98 (3)	Cl3^xii^—Dy—Na2^xiii^	64.54 (3)
Cl2^i^—Na1—Na2^ix^	52.04 (3)	Cl1^xii^—Dy—Na2^xiii^	44.32 (3)
Cl2^ii^—Na1—Na2^ix^	127.96 (3)	Cl1—Dy—Na2^xiii^	135.68 (3)
Cl3^iii^—Na1—Na2^ix^	137.55 (3)	Cl2—Dy—Na2^vi^	54.69 (3)
Cl3^iv^—Na1—Na2^ix^	42.45 (3)	Cl2^xii^—Dy—Na2^vi^	125.31 (3)
Cl1—Na1—Na2^ix^	106.72 (3)	Cl3—Dy—Na2^vi^	64.54 (3)
Cl1^v^—Na1—Na2^ix^	73.28 (3)	Cl3^xii^—Dy—Na2^vi^	115.46 (3)
Na2^vi^—Na1—Na2^ix^	107.98 (3)	Cl1^xii^—Dy—Na2^vi^	135.68 (3)
Na2^vii^—Na1—Na2^ix^	72.02 (3)	Cl1—Dy—Na2^vi^	44.32 (3)
Na2^viii^—Na1—Na2^ix^	180.0	Na2^xiii^—Dy—Na2^vi^	180.0
Cl1—Na2—Cl2^x^	97.84 (6)	Dy—Cl1—Na2	105.51 (5)
Cl1—Na2—Cl1^iv^	88.35 (5)	Dy—Cl1—Na2^vi^	95.24 (4)
Cl2^x^—Na2—Cl1^iv^	143.02 (7)	Na2—Cl1—Na2^vi^	133.80 (6)
Cl1—Na2—Cl3^i^	94.53 (6)	Dy—Cl1—Na1	134.58 (3)
Cl2^x^—Na2—Cl3^i^	79.84 (5)	Na2—Cl1—Na1	104.61 (4)
Cl1^iv^—Na2—Cl3^i^	136.23 (7)	Na2^vi^—Cl1—Na1	87.61 (4)
Cl1—Na2—Cl3^xi^	144.16 (7)	Dy—Cl2—Na1^xiv^	138.63 (3)
Cl2^x^—Na2—Cl3^xi^	74.54 (5)	Dy—Cl2—Na2^viii^	99.86 (4)
Cl1^iv^—Na2—Cl3^xi^	79.25 (5)	Na1^xiv^—Cl2—Na2^viii^	121.47 (5)
Cl3^i^—Na2—Cl3^xi^	117.64 (5)	Dy—Cl2—Na2^vi^	85.33 (4)
Cl1—Na2—Cl2^iv^	139.29 (7)	Na1^xiv^—Cl2—Na2^vi^	87.50 (4)
Cl2^x^—Na2—Cl2^iv^	119.28 (5)	Na2^viii^—Cl2—Na2^vi^	102.36 (4)
Cl1^iv^—Na2—Cl2^iv^	72.36 (4)	Dy—Cl3—Na1^vi^	134.93 (3)
Cl3^i^—Na2—Cl2^iv^	77.55 (4)	Dy—Cl3—Na2^xv^	128.25 (5)
Cl3^xi^—Na2—Cl2^iv^	68.06 (4)	Na1^vi^—Cl3—Na2^xv^	96.38 (4)
Cl1—Na2—Na1^iv^	104.40 (5)	Dy—Cl3—Na2^xi^	90.78 (4)
Cl2^x^—Na2—Na1^iv^	97.07 (5)	Na1^vi^—Cl3—Na2^xi^	82.33 (4)
Cl1^iv^—Na2—Na1^iv^	46.40 (3)	Na2^xv^—Cl3—Na2^xi^	104.74 (4)
Cl3^i^—Na2—Na1^iv^	161.07 (6)		

Symmetry codes: (i) *x*+1/2, −*y*+1/2, *z*+1/2; (ii) −*x*−1/2, *y*−1/2, −*z*+1/2; (iii) *x*−1/2, −*y*+1/2, *z*+1/2; (iv) −*x*+1/2, *y*−1/2, −*z*+1/2; (v) −*x*, −*y*, −*z*+1; (vi) −*x*+1/2, *y*+1/2, −*z*+1/2; (vii) *x*−1/2, −*y*−1/2, *z*+1/2; (viii) *x*−1, *y*, *z*; (ix) −*x*+1, −*y*, −*z*+1; (x) *x*+1, *y*, *z*; (xi) −*x*+1, −*y*, −*z*;  (xii) −*x*, −*y*, −*z*; (xiii) *x*−1/2, −*y*−1/2, *z*−1/2; (xiv) −*x*−1/2, *y*+1/2, −*z*+1/2; (xv) *x*−1/2, −*y*+1/2, *z*−1/2.
